# Chilblain lupus erythematosus associated with erdafitinib therapy in a patient with metastatic bladder cancer

**DOI:** 10.1016/j.jdcr.2024.10.007

**Published:** 2024-10-28

**Authors:** Amanda Truong, Jennifer Liu, Catherine Ni, Sharona Yashar

**Affiliations:** a Division of Dermatology, University of California, Los Angeles, Los Angeles, California; b Department of Dermatology, Veterans Affairs Greater Los Angeles Healthcare System, Los Angeles, California

**Keywords:** adverse reaction, chilblain lupus, cutaneous lupus, drug reaction, erdafitinib, oncodermatology, side effect, targeted therapy

## Introduction

The targeted therapy, erdafitinib, is a small-molecule inhibitor that has emerged as a pivotal second-line treatment for urothelial cancers harboring fibroblast growth factor receptor mutations. Notable cutaneous adverse events include nail abnormalities and hand-foot syndrome.^1^ Although reports of targeted therapies inducing autoimmune conditions are rare, here we present a case of a 57-year-old man who experienced chilblain lupus after erdafitinib treatment.

## Case report

A 57-year-old man, with a history of former tobacco use, seronegative inflammatory bowel disease-associated inflammatory arthritis and stage IV urothelial carcinoma, presented with painful necrosis of the distal fingers and toes. He was started on erdafitinib 6 months earlier after disease progression on avelumab (anti-programmed death-ligand 1 [PD-L1]). After 3 months of treatment, he presented with dystrophic nails and subsequent worsening onycholysis, erythema of the surrounding skin on the fingers and toes, and purulence of the bilateral feet. Podiatry performed nail avulsion and debridement. Despite infection resolution, he continued to have peeling and bleeding of his bilateral hands and feet. Two months later, imaging demonstrated decreased size of his metastases, but erdafitinib was stopped because of worsening cutaneous symptoms, including erosions and ulcerations, affecting his activities of daily living. He subsequently experienced necrosis of the distal fingers and toes over the next 2 weeks, prompting hospital admission for pain control and further workup.

**Fig 1:**
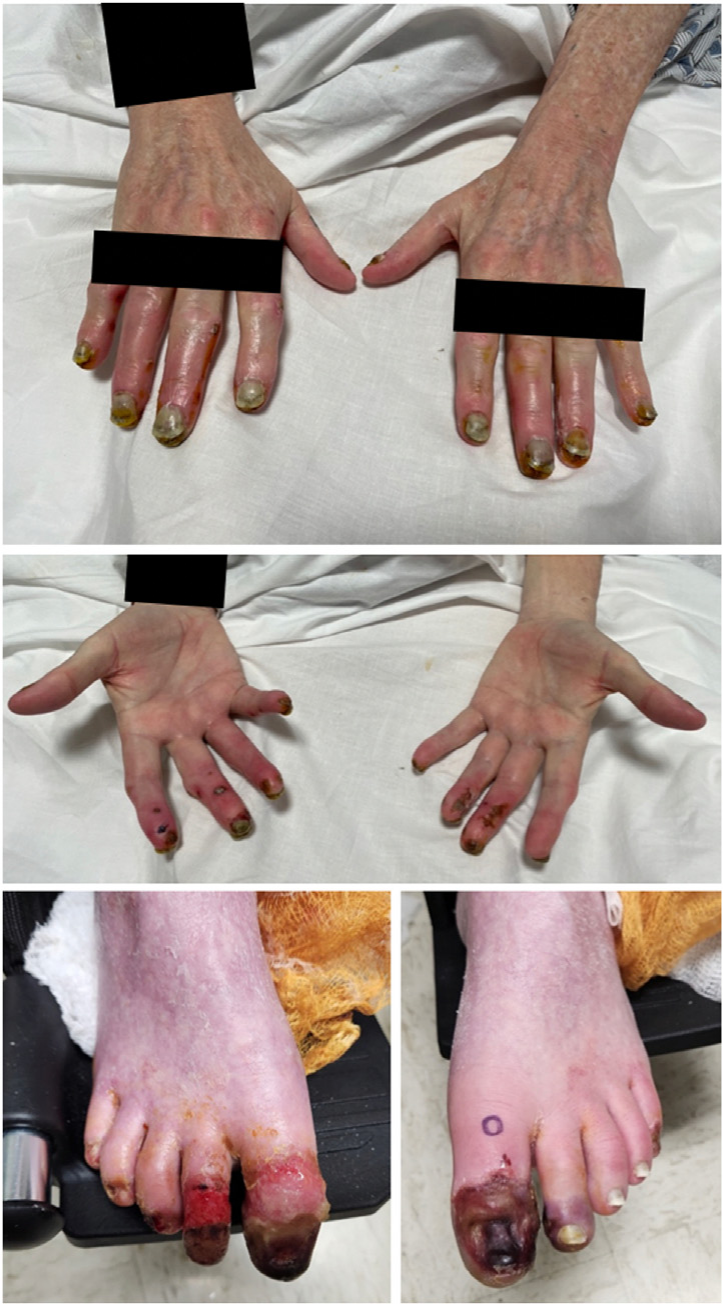
Chilblain lupus erythematous after erdafitinib as depicted by erythema and distal erosions and necrosis of the fingers and toes.

On physical examination, areas of necrosis were noted on all 10 distal fingertips, the first and second toes of the left foot, and first through third toes of the right foot, with surrounding scale and erythema. The fingernails exhibited hyperkeratotic subungual debris (Fig 1). No mucosal involvement was noted. The patient denied a history of Raynaud phenomenon, but did note worsening pain with colder temperatures. Initial laboratory workup revealed chronic normocytic anemia, but was otherwise unremarkable. Vascular workup, including coagulation panel, computed tomography angiogram of the chest, abdomen, and pelvis, upper-extremity ultrasound, and transthoracic echocardiogram, excluded a thromboembolic process. Rheumatologic workup was negative for antinuclear antibody, double-stranded DNA, ribonucleoprotein, histone, Ro, La, rheumatoid factor, cardiolipin antibody, β2-glycoprotein, lupus anticoagulant, antineutrophil cytoplasmic antibodies, and myeloperoxidase antibodies. In addition, cryocrit, serum protein electrophoresis, and urine protein electrophoresis were also negative.

**Fig 2:**
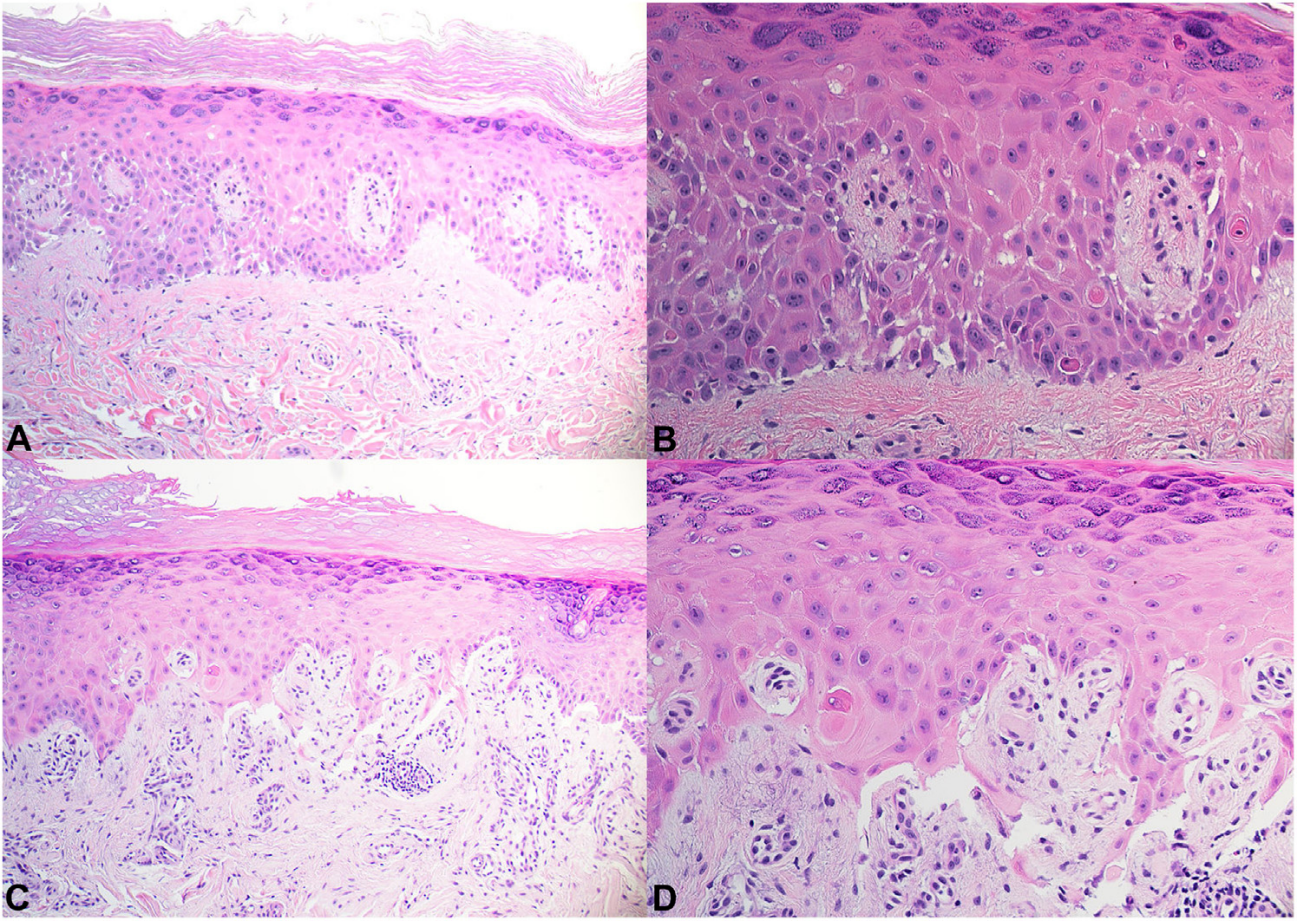
Histopathology of chilblain lupus shows interface dermatitis and spongiosis of the second finger of the right hand (**A, B**) and interface dermatitis, subepidermal split, small capillary proliferation with perivascular inflammation and endothelial cell damage of the first toe of the left foot (**C, D**).

A punch biopsy of erythematous skin on the second finger of the right hand showed interface dermatitis, with focal bullae, small capillary proliferation and endothelial cell damage (Fig 2, *A, B*). An additional punch biopsy of intact skin in the first toe of the left foot showed similar findings, both consistent with connective tissue disease (Fig 2, *C, D*). The clinical and histopathologic findings were most consistent with chilblain lupus.

**Fig 3:**
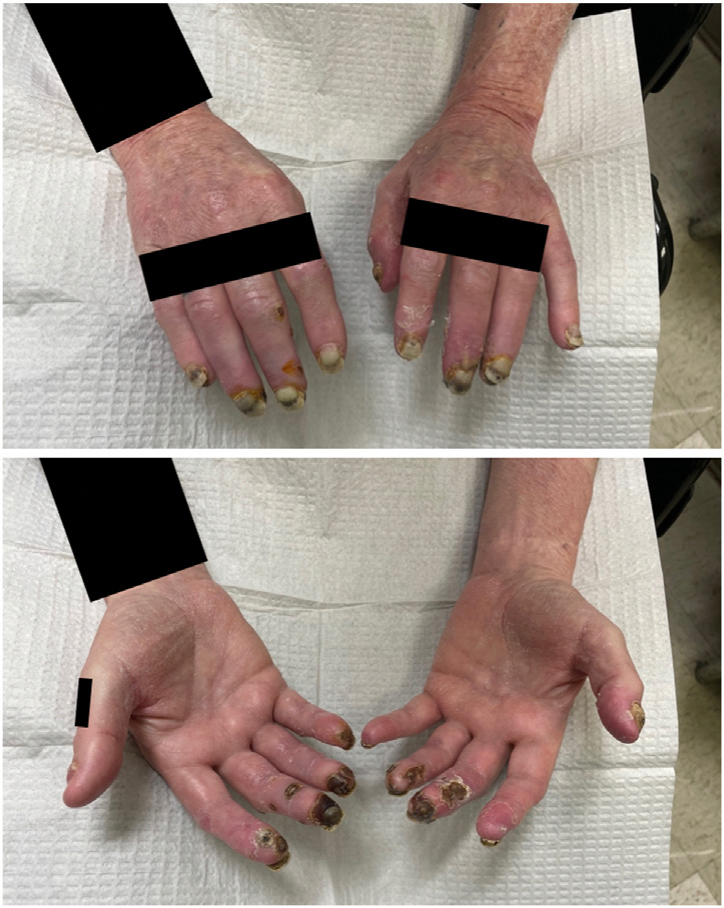
Improvement in erythema and necrosis of chilblain lupus erythematosus after 6 weeks of erdafitinib cessation and 3 weeks of topical calcium channel blocker.

The patient was initiated on topical nitroglycerin paste with minimal improvement after 1 week. Topical steroids were avoided because of risk of worsening vasoconstriction, and oral calcium channel blockers were contraindicated given hypotensive episodes while hospitalized. He was transitioned to topical diltiazem twice daily to erythematous areas and topical hydrophilic emollient to necrotic areas. Three weeks later (6 weeks after erdafitinib discontinuation), he had significant improvement in his pain and skin symptoms (Fig 3).

## Discussion

Targeted therapies provide protein-specific binding that confers advantages in inhibiting certain oncogenic drivers. Erdafitinib targets fibroblast growth factor receptors 2 and 3 and is a first-in-class medication that was recently Food and Drug Administration-approved for the treatment of locally advanced or metastatic bladder cancer in January 2024.^2^ In a phase 3 clinical trial, reported cutaneous side effects included hand-foot syndrome (30.4%), xerosis (23%), onycholysis (23%), onychomadesis (20.7%), nail dyspigmentation (17.8%), and alopecia (25.2%). Rheumatologic side effects were not reported.^1^

In addition to chilblain lupus, the clinical differential diagnosis for this patient’s presentation included thromboembolic disorders (including thromboangiitis obliterans, peripheral artery disease, and venous thromboembolism) and calciphylaxis. Thromboembolic events were excluded after extensive imaging, which is a critical initial step in the diagnostic process where distal necrosis is seen. Calciphylaxis was also considered, as it can present similarly and can be associated with rheumatologic conditions.^3^ However, this diagnosis was ruled out based on the patient’s reassuring laboratory findings, including normal kidney function, phosphorus level, parathyroid hormone level, calcium level, and vitamin D level, as well as the absence of calcium deposition or vessel lumen occlusion on histologic examination and lack of other risk factors, including concomitant anticoagulation. It is important to acknowledge, however, that the subcutaneous fat was not completely visualized in the histological samples, which could potentially affect the diagnostic certainty.

Chilblain lupus is a rare form of cutaneous lupus that commonly affects acral surfaces. Major diagnostic criteria include acral lesions associated with cold temperature or evidence of lupus erythematosus on histopathology. Additionally, minor criteria comprise coexistence with systemic lupus erythematous or other cutaneous lupus erythematosus, response to lupus therapy, or negative cryoglobulin or cold agglutinin studies.^4^ Treatment options include oral calcium channel blockers, topical vasodilators, systemic steroids, topical calcineurin inhibitors, or botulinum toxin injections.^5^^,^^6^ Topical steroids should be used with caution in necrotic lesions because of its exacerbation of vasoconstriction.^7^ For drug-induced causes, culprit drug cessation is crucial. In this case, although we largely attribute the patient’s clinical improvement to discontinuing erdafitinib, the addition of topical calcium channel blockade may also have contributed to some symptom control.

Although there have been no case reports to date of cutaneous lupus induced by erdafitinib, few case reports have reported inhibition of other receptor tyrosine kinases in triggering autoimmunity.^8^, ^9^, ^10^ The mechanism is not clearly understood, although may be related to aberrant inflammation after cell death induced by these therapies.^8^ Of note, this patient had a history of seronegative inflammatory bowel disease-associated inflammatory arthritis and prior antinuclear antibody positivity years prior, which may predispose him to autoimmunity. Nevertheless, here we report a novel case of chilblain lupus triggered by erdafitinib therapy.

## Conflicts of interest

None disclosed.
